# Ivabradine, atrial fibrillation and stroke: a combined meta-analysis and FAERS disproportionality analysis

**DOI:** 10.3389/fphar.2025.1638923

**Published:** 2025-11-19

**Authors:** Alberto Spadotto, Michele Fusaroli, Maria Carelli, Elena Nardi, Martina Amadori, Giulia Massaro, Veronica De Angelis, Milo Gatti, Valerio Ciubine, Emanuel Raschi, Elisabetta Poluzzi, Igor Diemberger

**Affiliations:** 1 Cardiology Unit, IRCCS Azienda Ospedaliero-Universitaria di Bologna, Bologna, Italy; 2 Department of Medical and Surgical Sciences, University of Bologna, Bologna, Italy; 3 Pharmacology Unit, Department of Medical and Surgical Sciences, University of Bologna, Bologna, Italy; 4 IRCCS Azienda Ospedaliero-Universitaria di Bologna, Bologna, Italy

**Keywords:** heart failure, atrial fibrillation, stroke, FAERS, diagnosis, heart rate, drug adverse event

## Abstract

**Background:**

Previous RCTs and meta-analyses observed an increased occurrence of atrial fibrillation (AF) associated with ivabradine use. Nonetheless, these studies were not focused on AF diagnosis, and it remains unclear whether this observed increase is due to a direct effect of ivabradine or just an augmented AF detection. The latter mechanism could arise from a greater heart-rate differential between sinus rhythm and AF under ivabradine, potentially intensifying symptoms and prompting earlier clinical evaluation. If this hypothesis is true, an earlier diagnosis of AF, and subsequent earlier prophylaxis with anticoagulants, may result in a reduced incidence of ischemic cerebrovascular events.

**Methods:**

We conducted a meta-analysis of the existing literature (calculating the ratio between ischemic cerebrovascular events and AF) combined with a disproportionality analysis of individual case safety reports of suspected adverse drug reactions. In the disproportionality analysis, we also included beta-blockers as a comparator group, given their dromotropic effect.

**Results:**

From 555 studies screened in the meta-analysis, only three were considered eligible. The ratio between ischemic cerebrovascular events and AF with ivabradine was lower than with placebo (RR 0.74, 95% CI 0.62–0.89; p < 0.001). In the FAERS, AF was disproportionally reported with both ivabradine and beta-blockers (Information Component 0.84, 95% CI 0.43–1.14 and Information Component 0.53, 95% CI 0.44–0.60), while ischemic cerebrovascular events only with beta-blockers (Information Component 0.25, 95% CI 0.18–0.31).

**Conclusion:**

Our findings raise the hypothesis that ivabradine facilitates an increased diagnosis rather than playing a direct role in causing AF. Prospective studies with continuous ECG monitoring and standardized endpoints are needed to clarify the temporal and mechanistic relationship between ivabradine, AF recognition, and cerebrovascular risk.

## 1 Introduction

Ivabradine exerts a dose-dependent reduction of the heart rate (HR) through selective and specific inhibition of hyperpolarization-activated cyclic nucleotide-gated (HCN) channels. HCN channels are responsible for the funny current (If) in the sinoatrial node, which influences the slope of the diastolic depolarization of the pacemaker action potential (DiFrancesco, 2006). Ivabradine is currently approved to improve clinical outcomes in patients with stable, symptomatic, chronic heart failure (HF) with reduced ejection fraction who are in sinus rhythm with resting heart rate ≥70 bpm who do not tolerate or have contraindications to beta-blockers (BB) or in combination with BB (McDonagh et al., 2021; Böhm et al., 2023; Marciniak et al., 2019). In addition, ivabradine was approved as a second-line therapy to relieve angina in adults with ischemic heart disease. Nevertheless, in the latest guidelines on chronic coronary syndromes, this indication has been restricted only to patients with reduced left ventricular ejection fraction (Knuuti et al., 2020; Vrints et al., 2024).

The most reported cardiovascular adverse effect of ivabradine is bradycardia (Fox et al., 2008; Fox et al., 2015). However, a more worrisome finding was the incidence of atrial fibrillation (AF) observed by the three major randomized controlled trials (RCTs) (Fox et al., 2008; Fox et al., 2014; Swedberg et al., 2010) and confirmed by meta-analyses, estimating an increase in the relative risk of AF of 15%–24% (Martin et al., 2014; Tanboğa et al., 2016; Wang et al., 2022). The risk of ischemic cerebrovascular events associated with AF may influence its use in clinical practice. Nonetheless, these studies were not focused on AF diagnosis, therefore, no specific monitoring approach was performed and the diagnosis was symptoms-driven, or carried out secondary to thromboembolic events, or during pre-specified follow-up. Furthermore, ivabradine’s higher specificity for the sinoatrial node, with minimal effect on the atrioventricular node (DiFrancesco, 2006; Fontenla et al., 2023), can increase the heart rate gap between sinus rhythm and AF, enhancing AF symptoms, and thus supporting its diagnosis. These two considerations raise the uncertainty that the higher rate of AF diagnosis may be driven by an increased perception rather than an increased incidence of AF in patients treated with ivabradine. To examine this hypothesis, we applied a triangulation strategy, integrating data from two independent sources in an exploratory framework.

## 2 Materials and methods

The preferential method to distinguish between the promotion of AF or an increased rate of diagnosis would require specific data on long-term ECG monitoring, comparing an active cohort treated with ivabradine and a control cohort. In the absence of these data in the available literature, we considered how this question could be explored with a different approach. Given the strong recommendation for oral anticoagulation in patients with AF and thromboembolic risk factors (i.e., coronary artery disease and HF), which antedates the enrollment in the RCT on ivabradine (Fuster et al., 2001; Lip et al., 2023), a new diagnosis of AF in these patients would hence have promoted a new prescription of oral anticoagulants. According to this consideration, an earlier diagnosis of AF, and the subsequent earlier prophylaxis with anticoagulants, may even imply a more effective prevention of stroke (Boriani et al., 2015; Schnabel et al., 2023; Fusaroli et al., 2024b). Viceversa, beta-blockers, which express a negative dromotropic effect on both the sinoatrial and atrioventricular nodes, could potentially mask AF and theoretically delay the initiation of anticoagulant therapy.

To make the underlying assumptions explicit and provide a more structured interpretation of the data, we developed conceptual Directed Acyclic Graphs (DAGs) illustrating two alternative and non-mutually exclusive scenarios: H_0_ (biological causation) and H_1_ (enhanced detection) (Tennant et al., 2021). These schematic representations were not intended for formal causal inference but rather to generate hypotheses and to visualize how different mechanisms could produce the observed associations between ivabradine exposure, AF reporting, and cerebrovascular events.

By outlining plausible relationships among clinical variables, confounders and mediators, the DAGs helped clarify which data patterns might be expected under each hypothesis. This conceptual exercise was not aimed at confirming or rejecting any causal pathway, but at guiding the interpretation of the results within a transparent framework that acknowledges uncertainty and the exploratory nature of the analysis, by helping to contextualize potential mechanisms and inform future hypothesis-driven investigations (Supplementary Figure S1).

Lacking current evidence to substantiate our hypothesis directly, available data were analyzed using two approaches: a) a meta-analysis of the scientific literature and clinical trials, and b) a disproportionality analysis of individual case safety reports (ICSRs) of suspected adverse drug reactions in the FDA Adverse Event Reporting System (FAERS) for ivabradine and beta-blockers.

### 2.1 Meta-analysis on incidence of AF and stroke

The systematic review was performed following the Preferred Reporting Items for Systematic Review and Meta-Analysis (PRISMA) guidelines. The study is registered at PROSPERO (CRD420251163238). The research question for the present review was constructed according to the PICO tool (Population, Intervention, Comparison, Outcome). In this study, the research question was whether ivabradine increases the risk of AF and potentially the risk of stroke, or whether ivabradine enhances the detection of AF, leading to potentially earlier prophylaxis with anticoagulants and a reduced incidence of ischemic cerebrovascular events. The following PICO format was applied: P: adults; I: ivabradine alone or in combination with standard care; C: standard care; and O: AF and ischemic cerebrovascular events occurrence. A systematic search using (MeSH)/Index terms was performed in PubMed, Embase, and ClinicalTrials.gov. The keywords used in the final search strategy were “Ivabradine or Procoralan or Corlentor” and the search was conducted until 1 September 2024. To ensure a reproducible approach, we applied specific inclusion and exclusion criteria to select relevant literature. We included only randomized controlled trials (RCTs), observational studies, and post-hoc analyses related to ivabradine use and reporting follow-up information on AF occurrence. After completing this initial screening, only studies reporting ischemic cerebrovascular events were considered. If ischemic cerebrovascular events or AF were not directly reported in the published report of the study, further information was retrieved from ClinicalTrials.gov (last access 1 October 2024). Considering the two sources of data, in the evidence of discrepancies between them, we performed a sensitivity analysis including only data from ClinicalTrials.gov to be compared to the original analysis mainly based on literature review. Studies with a sample size of less than 100 enrolled patients, enrolling patients <18 years old, reviews, posters, and case reports were excluded. The search was limited to studies in the English language. Duplicates were identified and removed via Covidence (version 2). An Excel form was prepared to support an objective and consistent extraction of the data. Different authors (A.S., M.A., M.C.) worked independently on the extraction of the data from eligible studies. The risk of bias was assessed by ROBINS-I tool (Sterne et al., 2016).

The primary endpoint of our analysis was the absolute incidence of AF and ischemic cerebrovascular events during follow-up and the ratio between stroke and AF incidence. Event counts for ischemic cerebrovascular events and AF were extracted from the included studies. Given the heterogeneity of the included trials (BEAUTIFUL, SHIFT, and SIGNIFY) in terms of study design and patient population, this synthesis was not intended to estimate a unified treatment effect but rather to explore the directional coherence between arrhythmic (AF) and ischemic cerebrovascular outcomes across distinct ivabradine-treated populations. The stroke-to-AF ratio and corresponding relative risks were calculated from crude event counts and are intended as descriptive indicators of coherence between arrhythmic and thromboembolic outcomes. Pooled RRs were calculated using both fixed effect (inverse variance weighted) and random effect (DerSimonian and Laird) models. Due to Statistical heterogeneity, the random effect model was preferred. Statistical heterogeneity across studies was quantified using the Cochran Q statistic and I2 statistic. The I2 statistic is derived from the Q statistic ([Q – df/Q]*100) and provides a measure of the proportion of the overall variation attributable to between-study heterogeneity. Given the small number of included trials (n = 3), the Egger’s test (Egger et al., 1997) was applied for completeness, but interpreted with caution, as publication bias assessments lack statistical reliability in such small samples. Two-sided P-values <0.05 were considered statistically significant.

### 2.2 Disproportionality analysis

FAERS is a global collection of individual case safety reports of suspected adverse drug reactions, submitted by consumers, healthcare professionals, and drug manufacturers. The reports are publicly available through an online Public Dashboard or by downloading the datasets as ASCII or XML files, which are published quarterly. We used access to a pre-processed version of the FAERS database provided by the DiAna R package, which, among other advantages, maps drug names recorded as free text to active ingredients using a manually curated dictionary (DiAna Dictionary) (Fusaroli et al., 2024a). FAERS is a well-established pharmacovigilance database with global real-world data and proved to be valuable in identifying and evaluating cardiovascular safety signals, complementing RCT data (Gatti et al., 2021).

The disproportionality analysis on ivabradine was conducted by retrieving relevant datasets from the FAERS database compiled between the first quarter of 2004 (2004 Q1) and the first quarter of 2023 (2023 Q1). The events of interest – AF and ischemic cerebrovascular events – were retrieved using preferred terms (PTs) in the Medical Dictionary for Regulatory Activities (MedDRA) Dictionary (Version 26.0) following the inherent Standardized MedDRA Queries (SMQ) (see the Supplementary Material for a list of PTs used to retrieve these suspected reactions, Supplementary Table S1). To remove duplicates, we considered only once reports with the same value in all the following fields: gender, age, country, event date, drug list, and event list.

Based on the reported indications for ivabradine use (Supplementary Table S2 in Supplementary Material), we identified three study populations: patients with heart failure (HF), patients with ischemic heart disease (IHD), and any of the two conditions (IHD_HF) to take into account confounding by indication. It is important to clarify that the most recent guidelines for patients with chronic coronary syndromes have restricted the indication for ivabradine to those with a left ventricular ejection fraction below 40% (Vrints et al., 2024). Since the study was conducted using FAERS data up to the first quarter of 2023, the analysis was based on the indications that were in effect until that time, which do not impose a restriction associated with ejection fraction (Knuuti et al., 2020). An additional analysis was performed considering only reports that occurred after the approval of ivabradine (IHD_HF post-approval) to take into account potential confounding by different time on the market.

The Information Component (IC) was used as a measure of disproportionality, comparing the proportion of reports recording the event of interest among the reports recording the drug of interest with the proportion of reports recording the same event among the reports recording other drugs (Evans et al., 2001). Specifically, IC was calculated as

$$IC\;\left(x,y\right)=\log_{2}\frac{p\;\left(y,x\right)}{p\left(x\right)p\left(y\right)}=\log_{2}\frac{a*N}{\left(a+b\right)*\left(a+c\right)}\approx \log_{2}\frac{a+0.5}{\frac{\left(a+b\right)*\left(a+c\right)}{N}+0.5}$$

where “a” is the number of patients developing a target event (AF or ischemic cerebrovascular event) when they received a target drug (ivabradine, bisoprolol, metoprolol, atenolol, carvedilol or nadolol), “b” is the number of patients developing non-target adverse events with the target drug, “c” is the number of patients developing the target event when they received non-target drugs, and “d” is the number of patients developing non-target adverse events when they received non-target drugs (Cutroneo et al., 2024).

A significant association was identified when the entire 95% confidence interval of the IC was above 0. Using disproportionality analyses to compare hypotheses is a non-traditional approach that seeks to go beyond conventional signal detection.

Of note, the FAERS analysis was performed as an exploratory disproportionality assessment intended to generate and contextualize safety signals against clinical trial evidence, acknowledging that spontaneous report data cannot support causal inference. The reporting of FAERS analysis adhered to the READUS-PV Guidelines, The REporting of A Disproportionality analysis for drUg Safety signal detection using individual case safety reports in PharmacoVigilance (Fusaroli et al., 2024c).

#### 2.2.1 Disproportionality analysis on beta-blockers

Beta-blockers exert a negative dromotropic effect on both the sinoatrial and atrioventricular nodes (Wołowiec et al., 2023), which may attenuate AF symptoms and theoretically delay recognition and the initiation of anticoagulation therapy. To explore this pharmacological contrast with ivabradine, the FAERS analysis was extended to include beta-blockers as external comparators, both as a drug class and as five individual agents (atenolol, bisoprolol, carvedilol, metoprolol, and nadolol). Although purely hypothesis-generating, this disproportionality approach enables a comparison between ivabradine and beta-blockers and the investigation of a potentially opposite effect on AF detection.

## 3 Results

### 3.1 Meta-analysis on incidence of AF and stroke

**FIGURE 1:**
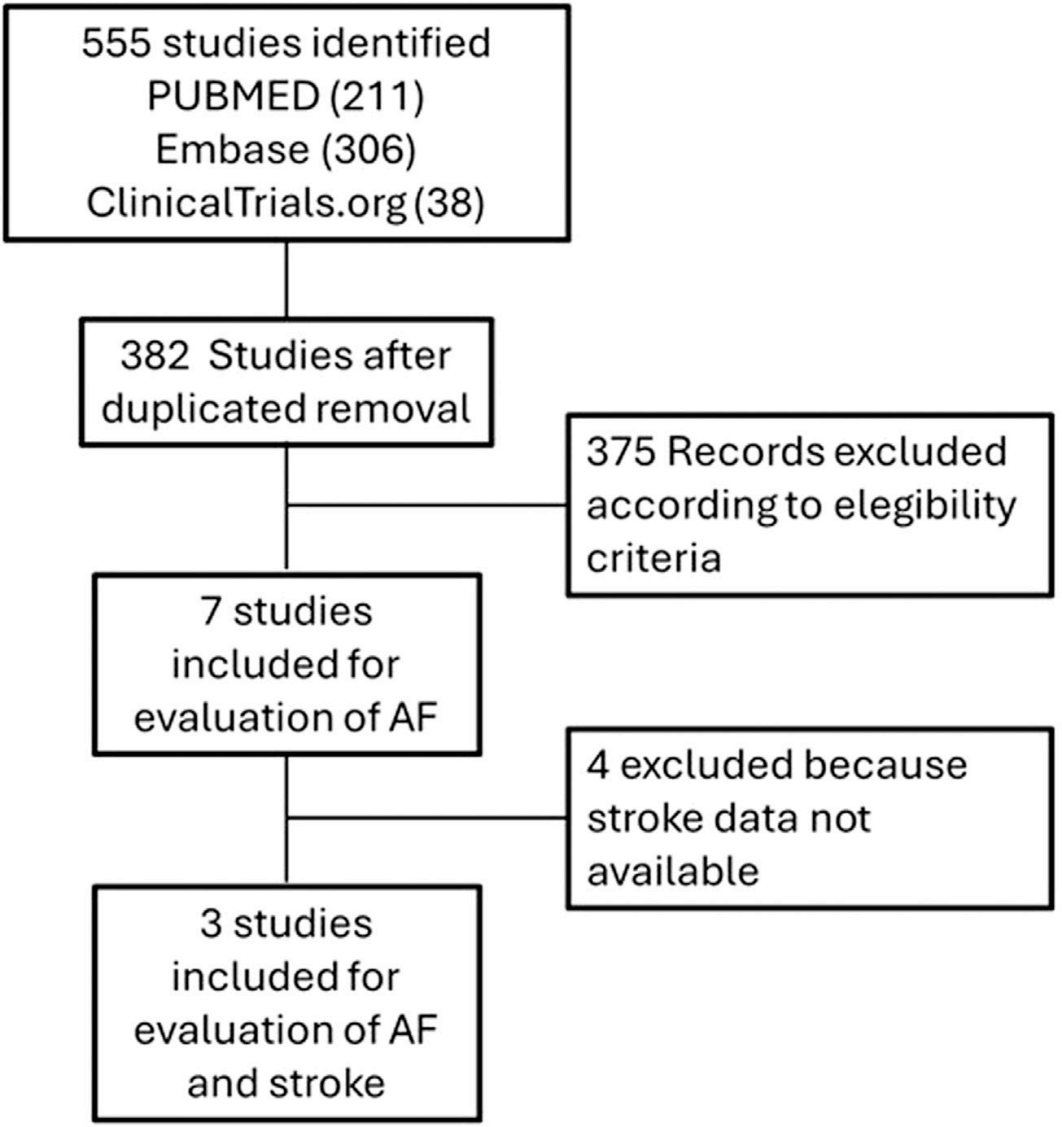
Flow chart of study selection.

A total of 382 studies out of 555 were selected after removing duplicates. Among these, seven studies met the eligibility criteria by reporting information about the occurrence of AF. Among these seven studies, three (Ivabradine for patients with stable coronary artery disease and left-ventricular systolic dysfunction [BEAUTIFUL]: a randomized, double-blind, placebo-controlled trial (Fox et al., 2008); Ivabradine and outcomes in HF [SHIFT]: a randomized placebo-controlled study (Swedberg et al., 2010); Ivabradine in stable coronary artery disease without clinical HF [SIGNIFY] (Fox et al., 2014)) provided data on the incidence of both AF and ischemic cerebrovascular events (data on ischemic cerebrovascular events were available only for SIGNIFY trial, on the other case they were retrieved from ClinicalTrials.gov). The flow chart for studies selection is reported in Figure 1, the characteristics of the studies and the reported adverse events are described in Table 1 and Supplementary Table S3.

**TABLE 1:** Studies included in the meta-analysis and reported events (the number of events was obtained directly from the trial or, when not available, from ClinicalTrials.gov).

Study	Design	Year	Intervention	Number of patients	Ivabradine		Placebo		Median follow-up
					AF	Stroke	AF	Stroke	
BEAUTIFUL (Fox et al., 2008)	RCT	2008	Iva vs. Pla	5,477 vs. 5,430	334	107	310	119	19 months
SHIFT (Swedberg et al., 2010)	RCT	2010	Iva vs. Pla	3,241 vs. 3,264	306	68	251	94	22.9 months
SIGNIFY (Fox et al., 2014)	RCT	2014	Iva vs. Pla	9,539 vs. 9,544	508	162	362	151	27.8 months

AF, atrial fibrillation; Iva, ivabradine; RCT, randomized clinical trials; Pla, placebo.

**FIGURE 2:**
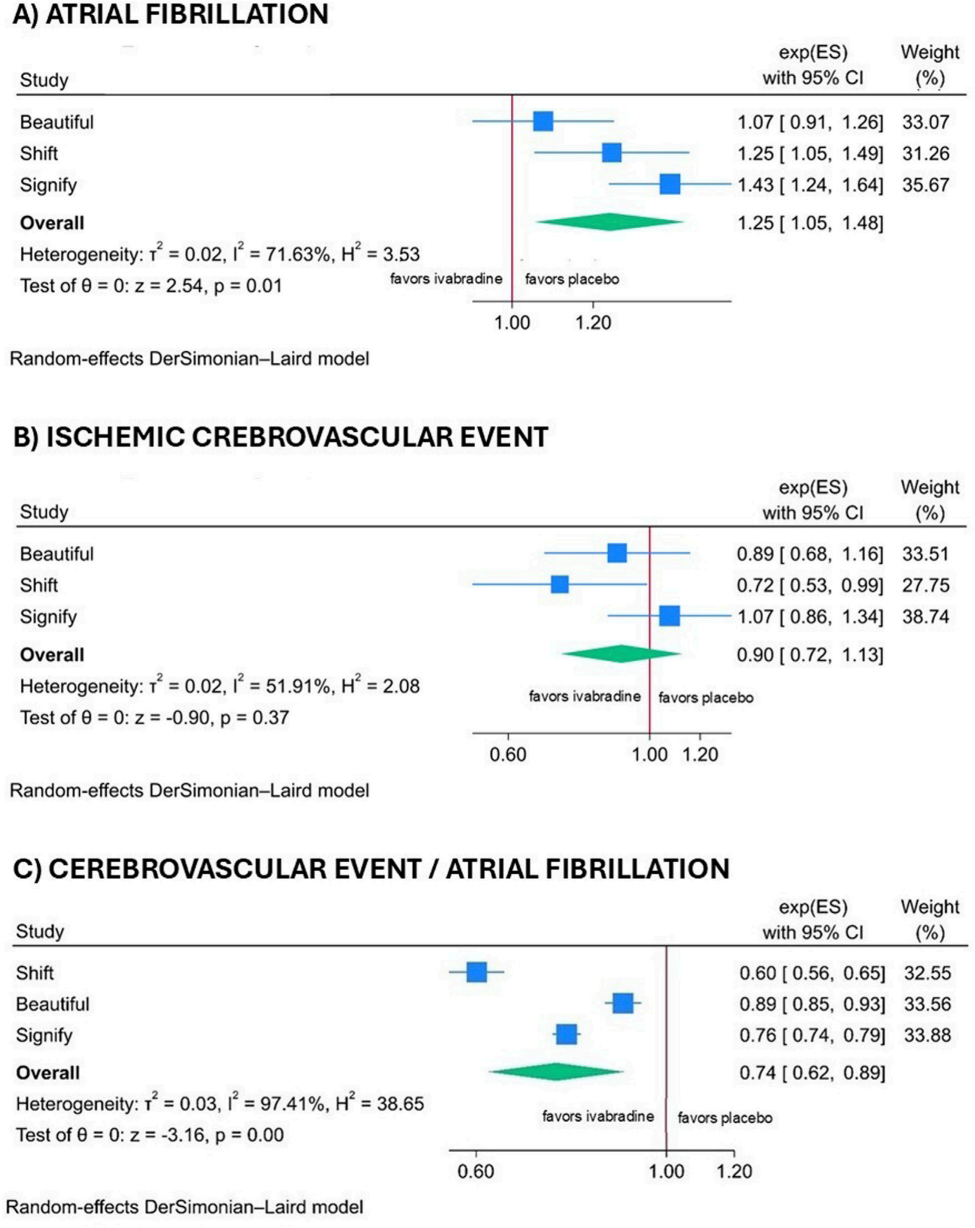
Forest plot illustrating the atrial fibrillation **(A)**, the cerebral ischemic events **(B)** and the ratio of cerebral events to atrial fibrillation **(C)** across all trials, using clinical study data when available, and otherwise utilizing data from ClinicalTrials.gov.

Ivabradine was associated with a higher incidence of AF compared with the control group (RR 1.25; 95% CI 1.05–1.48; p = 0.01); however, this increase did not translate into a higher rate of stroke (RR 0.90; 95% CI 0.71–1.13; p = 0.37). The ratio between cerebrovascular events and AF demonstrated a significant difference between the ivabradine and control groups (RR 0.74; 95% CI 0.62–0.89; p < 0.001), as illustrated in the forest plot in Figure 2. No publication bias was detected (Egger’s test p = 0.34) (Supplementary Figure S3 in the Supplementary Material). The results of the sensitivity analysis using only data on AF and ischemic cerebral events obtained from ClinicalTrials.gov are comparable and available in the Supplementary Material, Supplementary Table S4 and Supplementary Figure S3.

### 3.2 Disproportionality analysis

**FIGURE 3:**
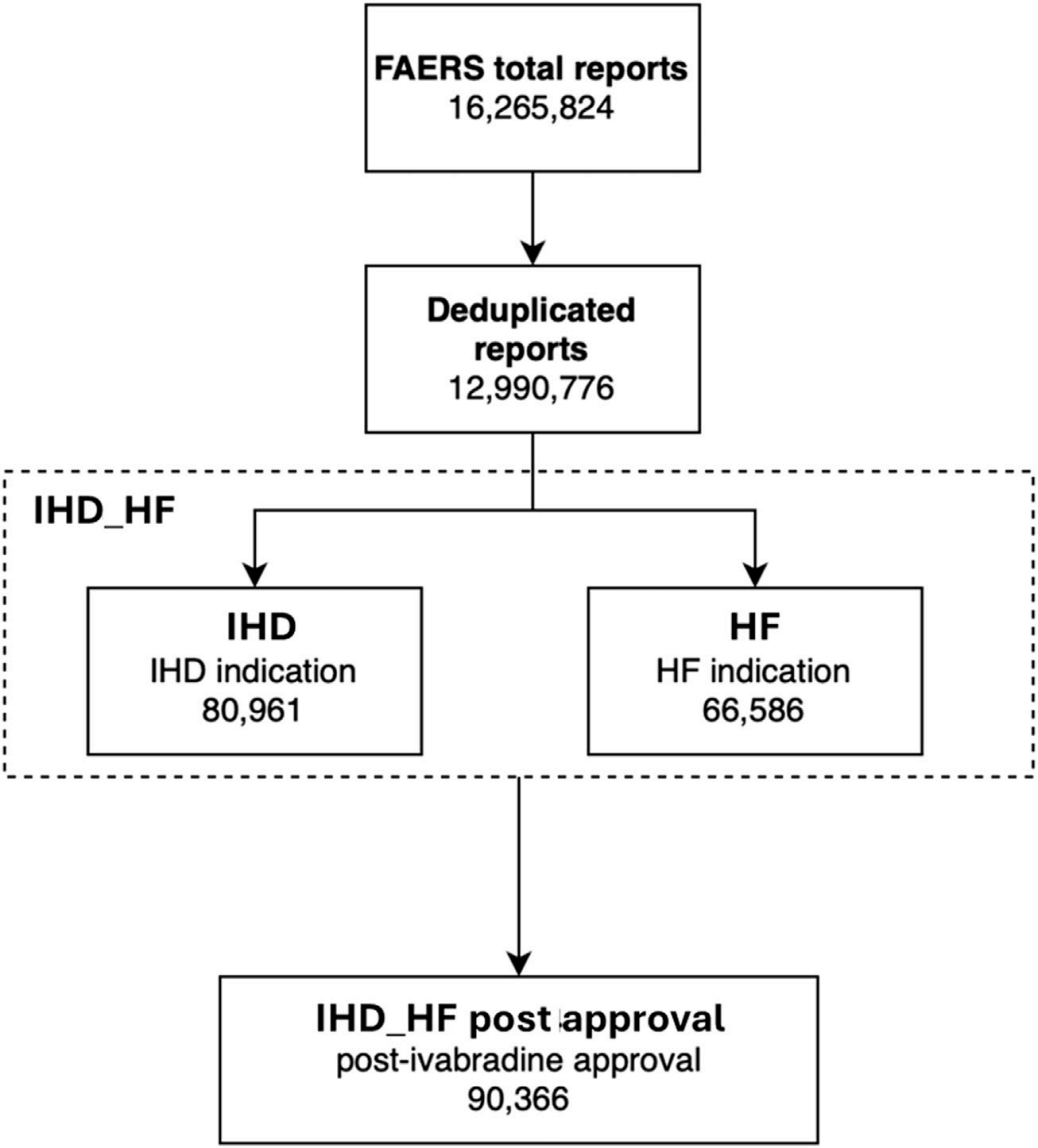
Groups analyzed in the FAERS analysis. HF, heart failure; IHD, ischemic heart disease; IHD_HF, ischemic heart disease and/or heart failure.

We retrieved a total of 16,265,824 ICSRs from the FAERS database up to 2023 Q1. After deduplication, 12,990,776 cases were analyzed. We identified 8,026, 130,639, 138,911, 126,455, 341,421, and 7,961 reports recording any adverse events while receiving ivabradine, atenolol, bisoprolol, carvedilol, metoprolol, and nadolol, respectively. The number of reports based on the different populations considered is summarized in Figure 3.

The number of cases for reported adverse events and the IC confidence intervals for AF PT and stroke PT in the different population subgroups are summarized in the Supplementary Material, Supplementary Table S5.

**FIGURE 4:**
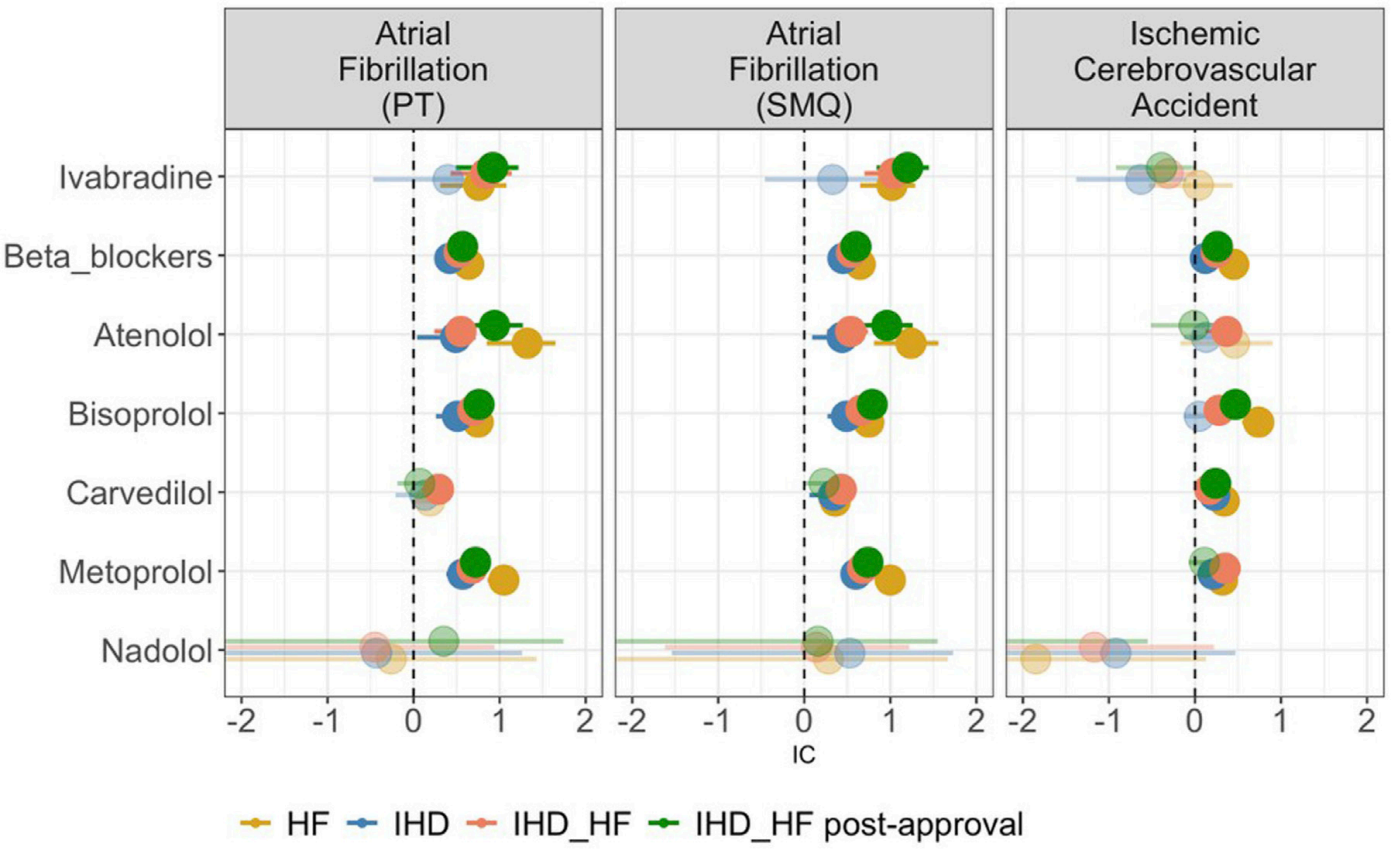
Forest plot of the Information Component (IC) of ivabradine, beta-blockers, and single beta-blocker molecules (atenolol, bisoprolol, carvedilol, metoprolol, and nadolol) in the different study populations. The circles represent medians, with sizes proportional to the number of cases. The line represents the 95% confidence interval. Transparent circles indicate the absence of a signal, and if the confidence interval is missing, it means that the lower limit is less than 0. HF, heart failure; IHD, ischemic heart disease; IHD_HF, ischemic heart disease and/or heart failure.

Figure 4 depicts the results of disproportionality analyses. Considering the HF group, both the beta-blocker class and ivabradine are disproportionally reported with AF. Analyzing individual beta-blockers, this finding is confirmed for metoprolol, atenolol, and bisoprolol, but not for carvedilol and nadolol. When considering the IHD population, this disproportion manifests for the beta-blocker class, but not for ivabradine. When considering IHD_HF, there is a significant disproportionate reporting of AF for both ivabradine and beta-blockers (including individual molecules, except for nadolol), and this was confirmed in the subgroup analysis of IHD_HF post-approval (except for carvedilol).

When considering disproportionate reporting for ischemic cerebrovascular events in the HF group, there is a significant disproportionate reporting for beta-blockers as a class, as well as for individual molecules (except for atenolol and nadolol). However, no disproportion is observed for ivabradine (IC 0.04, 95% CI -0.54 – 0.44). There is no disproportionate reporting for ivabradine in the IHD, IHD_HF and IHD_HF post-approval group either. In these three populations, the disproportionality is confirmed in beta-blockers as a class.

The analyses performed using the AF SMQ are comparable to those using AF PT and are reported in the Supplementary Material (Supplementary Table S6).

## 4 Discussion

The observation of an increased rate of AF among patients receiving ivabradine has prompted ongoing debate regarding whether this reflects a true pharmacologic effect or increased clinical recognition. To clarify this issue, we combined evidence from a meta-analysis and FAERS disproportionality analysis. Our results consistently show an association between ivabradine and AF, but not with ischemic cerebrovascular events. These findings suggest that the reported higher incidence of AF with ivabradine may not represent a true increase in AF occurrence, but rather reflect enhanced detection or earlier recognition of previously unrecognized episodes, generating the hypothesis that ivabradine may facilitate AF diagnosis and not the overall AF incidence. Of note, previous studies (Fox et al., 2008; Fox et al., 2014; Swedberg et al., 2010; Martin et al., 2014; Tanboğa et al., 2016; Wang et al., 2022) which identified a statistical association between recorded AF and ivabradine were not specifically designed to detect either AF episodes or, more appropriately, AF burden. Indeed, adequate detection of these endpoints would entail additional assessments such as prolonged ambulatory ECG monitoring, implantable loop recorders, or at least periodical pulse self-assessment. (Kalarus et al., 2023; Van Gelder et al., 2024). Under this design, AF diagnosis was obtainable only in case of symptoms (leading to hospital admission or identified through clinical interviews by investigators involved in the RCT), thromboembolic events, or pre-specified follow-up. Since any factor that increases the occurrence or severity of AF symptoms (e.g., palpitations) would prompt earlier medical contact, thereby increasing the likelihood of AF detection and earlier initiation of oral anticoagulation in otherwise asymptomatic patients. Ivabradine, by reducing heart rate during sinus rhythm, may enhance symptom perception at the onset of AF and therefore increase the likelihood of AF diagnosis without necessarily raising its true incidence. Although speculative, ivabradine might confer some protection against stroke by facilitating earlier detection of AF, a mechanism that could reduce the burden of unrecognized and therefore untreated AF, which is associated with increased cerebrovascular risk. (Boriani et al., 2015; Christow et al., 2017; Lin et al., 2023). Interestingly, in the data reported in a subanalysis of the SIGNIFY trial, which analyzed the incidence of fatal and non-fatal strokes in patients with and without AF, no variation in the percentage of strokes was observed between patients treated with ivabradine versus placebo (1.7% vs. 1.6%) (Fox et al., 2015). Likewise, analyzing the incidence of both AF and stroke in the meta-analysis of available RCTs, we observed an increased diagnosis of AF without a concomitant rise in ischemic cerebrovascular events, expected complications of AF. By contrast, in studies investigating drugs suspected to play a causal role in the development of AF, such as ibrutinib, patients exposed to the drug demonstrate an increased risk of AF that is paralleled by a higher incidence of ischemic cerebrovascular events. This pattern emerges both in a cohort study and in pharmacovigilance analyses, where a disproportionality signal for reports of both AF and ischemic stroke has been documented (Diamond et al., 2023; Zheng et al., 2023).

Our findings are relevant for two considerations: (a) the prescription of ivabradine can be affected by the reported increased incidence of AF (Fox et al., 2008; Fox et al., 2014; Swedberg et al., 2010) and (b) they may provide new insight into a long-standing controversy surrounding the use of anticoagulation in patients with HFrEF and sinus rhythm (Doehner et al., 2023). As shown by two recent meta-analyses on WASH, HELAS, WATCH, WARCEF, and COMMANDER-HF trials, the use of oral anticoagulation consistently reduced stroke risk in HFrEF and sinus rhythm (Beggs et al., 2019; Zhang et al., 2020). Beyond the explanations provided by the authors for the higher risk of stroke in HF patients (e.g., hypercoagulation state due to higher platelet activity and thrombin generation, endothelial dysfunction, reduction in intracardiac flow due to reduced left ventricle enlargement and reduced contractility), our findings may suggest an additional contributing factor. Specifically, the rate-controlling properties of beta-blockers commonly used in HFrEF might delay or mask the recognition of AF episodes, thereby leading to underdiagnosis and, consequently, suboptimal stroke prevention.

Despite these efforts, our approach is still limited by the lack of patient-level temporal information linking AF and stroke within the same individual. As detailed in the next section, in the studies included in our meta-analysis many patients received ivabradine in addition to a beta-blocker rather than as an alternative, which may affect the overall interpretation of the findings, although ivabradine’s heart rate–lowering effect in sinus rhythm is unlikely to be impacted.

To mitigate design-related constraints, we incorporated a second source of evidence by including the FAERS evaluation. This approach enabled assessment of consistency across different sources, by assessing disproportionate reporting of AF and ischemic cerebrovascular events associated with ivabradine use. Furthermore, it provided the opportunity to contrast reporting patterns with beta-blockers, a comparison not fully addressed in the existing literature (not possible in RCTs since, to our knowledge, there are no comparative studies between ivabradine and beta-blockers, except potentially in the acute setting) (Iliuta and Rac-Albu, 2014). The results of the FAERS analysis are consistent with our meta-analysis, evidencing a significant disproportion in the reporting of AF with both ivabradine and beta-blockers, whereas disproportionate reporting of stroke was observed only with beta-blockers. At this regard, Lindholm et al. (Lindholm et al., 2005) reported a higher risk of stroke in over 100,000 patients treated for primary hypertension with beta-blockers compared to other antihypertensive agents. This phenomenon seems even more pronounced in patients over 60 years of age (Khan, 2006) and among those treated with atenolol leading to the concept that the increased arterial stiffness and pulse wave dyssinchrony, characterizing systemic hypertension in the elderly, may be less responsive to beta-blockers. Nevertheless, this interpretation has already been questioned by some authors (Khan, 2006; Kuyper and Khan, 2014), and is further challenged by our FAERS analysis, which suggests a broader class effect. Taken together, this pattern may be consistent with our proposed explanation for the seemingly higher occurrence of AF in ivabradine-treated patients, reflecting differential effects of ivabradine and beta-blockers on the sinus and atrioventricular nodes (DiFrancesco, 2006). Consistent with this mechanistic interpretation, current guidelines recommend beta-blockers as first-line agents for rate control in AF (Van Gelder et al., 2024), especially in patients with HF, while preliminary studies on the efficacy of ivabradine as a rate-control agent so far reported discouraging results (Fontenla et al., 2023). Nevertheless, ivabradine could theoretically anticipate the diagnosis of AF, possibly leading to earlier anticoagulation. Of note, this mimics the finding on the effect of oral anticoagulants in reducing cancer-related mortality, which may have been mediated by an earlier diagnosis of colorectal cancers (Andreotti and Maggioni, 2022).

## 5 Strengths and limitations

This study integrates evidence from two different sources, randomized controlled trials and pharmacovigilance data (individual case safety reports from FAERS), to explore the relationship between ivabradine, AF, and ischemic cerebrovascular events. The main strength lies in this hypothesis-generating framework, combining a quantitative synthesis of clinical trials with a large-scale FAERS disproportionality analysis to test epidemiological coherence. The consistent pattern observed across these independent data sources strengthens the internal validity of our observations and supports the plausibility of a mechanism involving facilitated AF detection rather than AF induction.

However, several limitations must be acknowledged. First, the meta-analysis is based on a small number of trials (BEAUTIFUL, SHIFT, and SIGNIFY) that differ in design, population, and endpoint definitions. This may bias effect estimates toward overestimating coherence between AF and stroke, as the trials were not powered for these outcomes. Moreover, AF and stroke were secondary or exploratory outcomes in the included trials, so the stroke-to-AF ratio, used as a conceptual indicator to explore coherence between arrhythmic and thromboembolic outcomes, albeit interesting, should not be interpreted as a causal metric. Stroke-to-AF ratio could potentially be confounded by differences in anticoagulation use, stroke subtype, detection bias, and baseline cardiovascular risk. The lack of patient-level temporal information further limits causal inference, as the temporal sequence between AF and stroke cannot be verified in these datasets. Notably, so far, evidence of a temporal relationship between AF episodes and ischemic stroke remains incompletely understood. Whether this is due to a true absence or due to varying study methodology, lack of control for anticoagulation or inconsistent stroke subtyping remains to be established (Dlima et al., 2024).

In addition, while the FAERS analysis offers a valuable additional perspective, it is subject to reporting biases such as underreporting, notoriety bias (particularly after publications linking ivabradine to AF), confounding by indication, missing data and lack of denominator (population exposure) and clinical data (no access to narratives), which do not allow to infer causality, to estimate rates or incidence. Therefore, the comparison with beta-blockers must be regarded as exploratory.

Of note, studies providing patient-level data are currently lacking, and their implementation would face considerable methodological and financial challenges. Ivabradine is indicated for selected populations with HF or stable coronary disease, in whom long-term continuous ECG monitoring would be logistically complex and potentially unjustified in the absence of a specific indication, with potential issues connected to identification of minor ECG findings (e.g., short high rate atrial/ventricular episodes). Despite these challenges, such studies would be invaluable to validate the present findings and elucidate the causal structure of ivabradine-related AF, further strengthening the triangulation of evidence across multiple data sources.

## 6 Conclusion

In conclusion, we presented a set of findings spanning adverse event reports, meta-analysis of the scientific literature, and biological plausibility, which collectively challenge the commonly held, yet weakly substantiated, belief that ivabradine directly promotes AF. Instead, our results raise an alternative hypothesis: that ivabradine may facilitate early AF diagnosis by amplifying AF-related symptoms, due to a relatively higher gap in heart rate between AF and sinus rhythm. If confirmed, this could ultimately result in earlier control of the risk of thromboembolism and stroke. This potential mechanism, while biologically plausible, remains speculative and cannot be confirmed with the available data. Our results should therefore be interpreted as exploratory and hypothesis-generating, highlighting the need for prospective studies incorporating continuous ECG monitoring and standardized outcome definitions to test the proposed causal framework and clarify the temporal and mechanistic links between ivabradine, AF diagnosis, and cerebrovascular risk.

## Data Availability

The original contributions presented in the study are included in the article/Supplementary Material, further inquiries can be directed to the corresponding author.
