# Supplementary material for: Ivabradine, atrial fibrillation and stroke: a combined meta-analysis and FAERS disproportionality analysis
Source: Front Pharmacol. 2025 Nov 19;16:1638923. doi: 10.3389/fphar.2025.1638923 (PMC12672418; doi:10.3389/fphar.2025.1638923)

Supplementary Material

PUBMED

("ivabradine"[MeSH Terms] OR "ivabradine"[MeSH Terms] OR "ivabradine"[All Fields] OR "ivabradin"[All Fields] OR "ivabradine s"[All Fields] OR "corlentor"[All Fields] OR "procoralan"[All Fields]) AND (clinicaltrial[Filter] OR randomizedcontrolledtrial[Filter])

EMBASE

('ivabradine'/exp OR ivabradine) AND ([controlled clinical trial]/lim OR [randomized controlled trial]/lim)

**Table S1**

List of PTs used to retrieve these suspected reactions

1. Supraventricular tachyarrhythmias (SMQ)


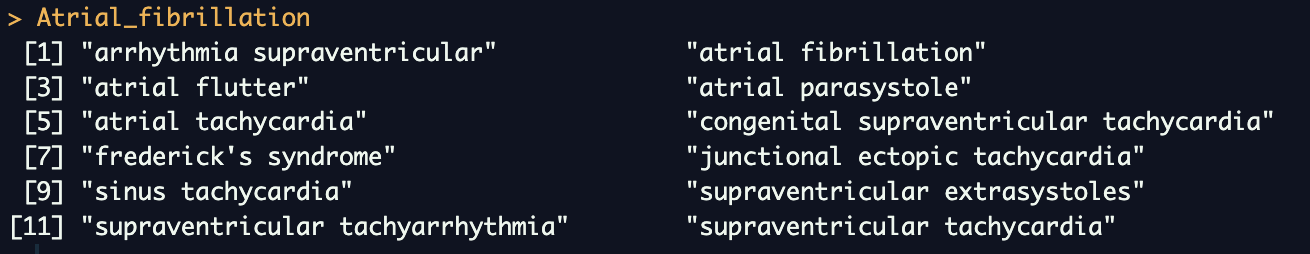


1. Ischemic central nervous system vascular conditions (SMQ)
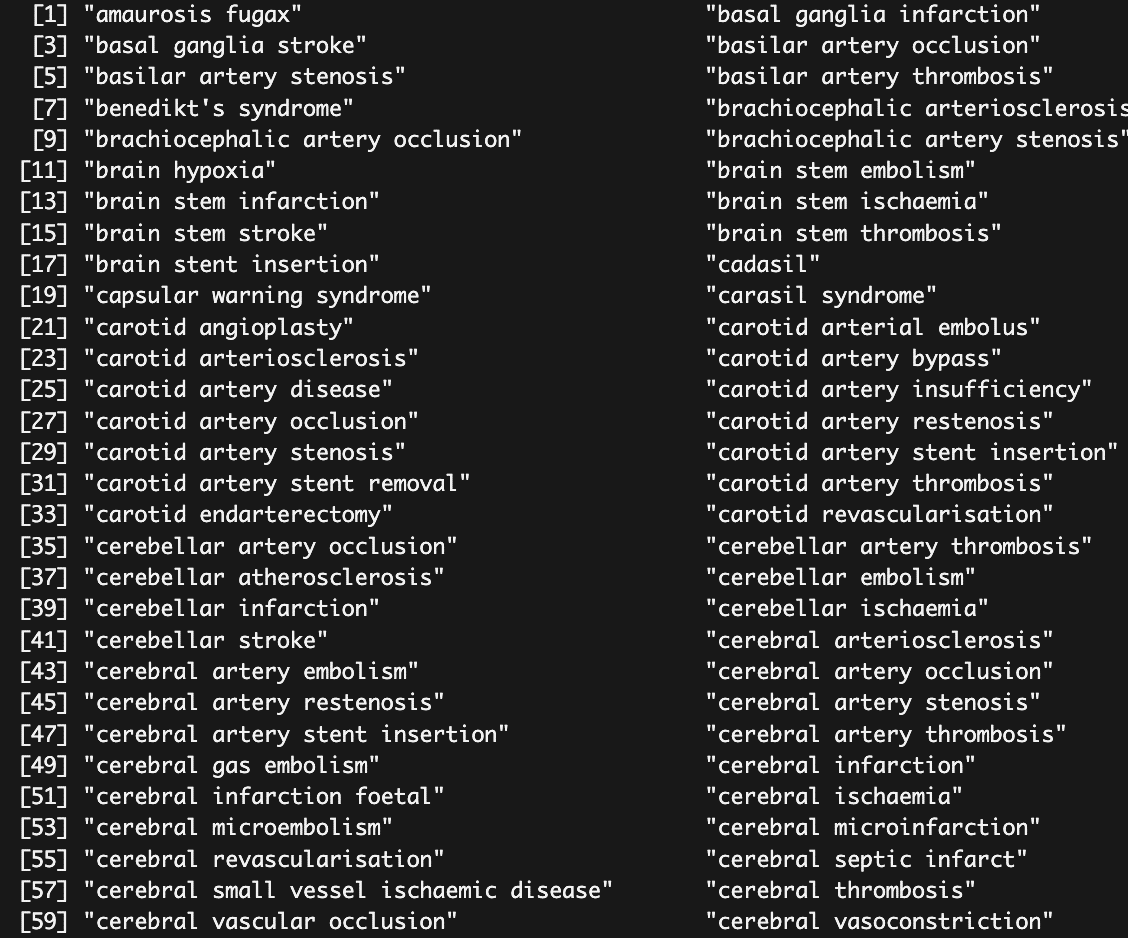

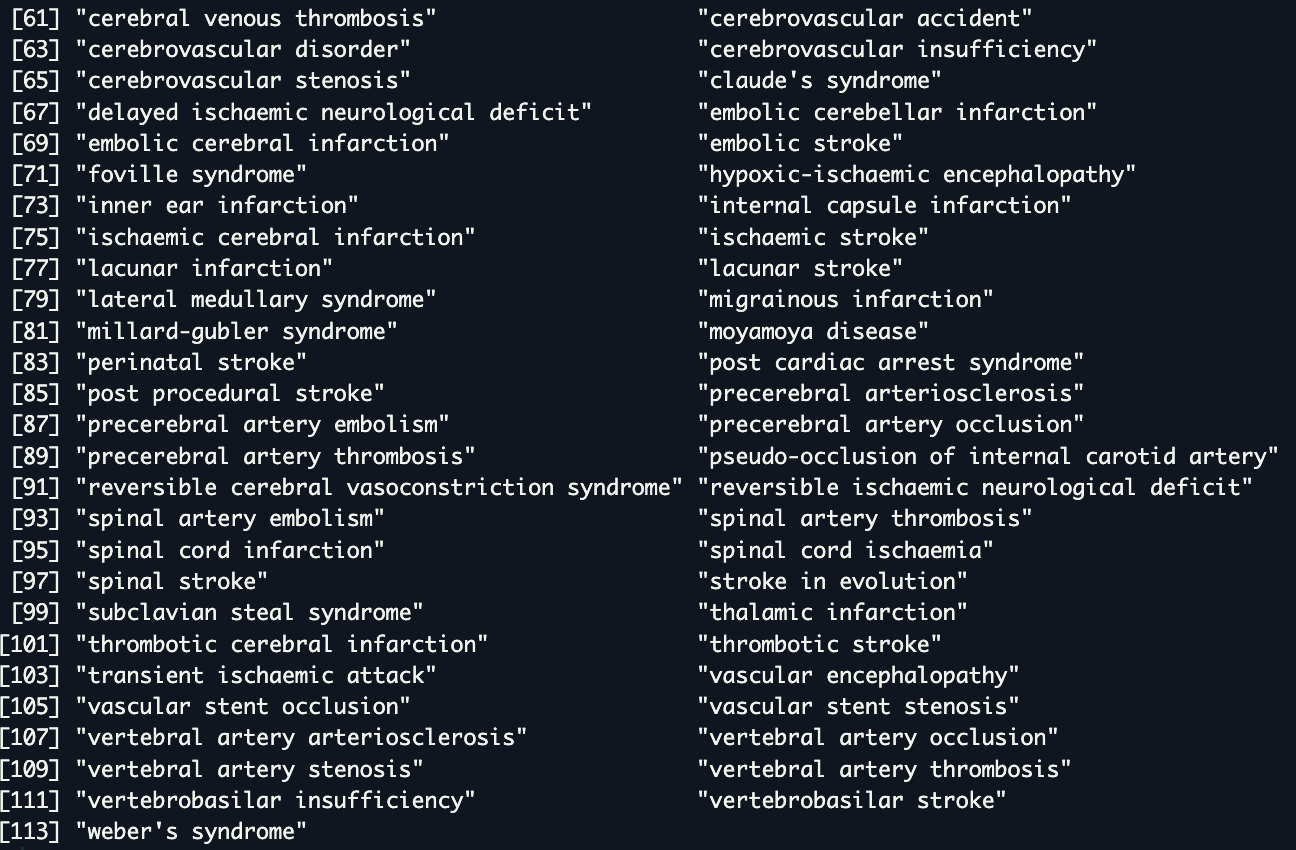


**Table S2**

List of PTs used to retrieve these approved indications

1. Ischemic heart disease (SMQ)


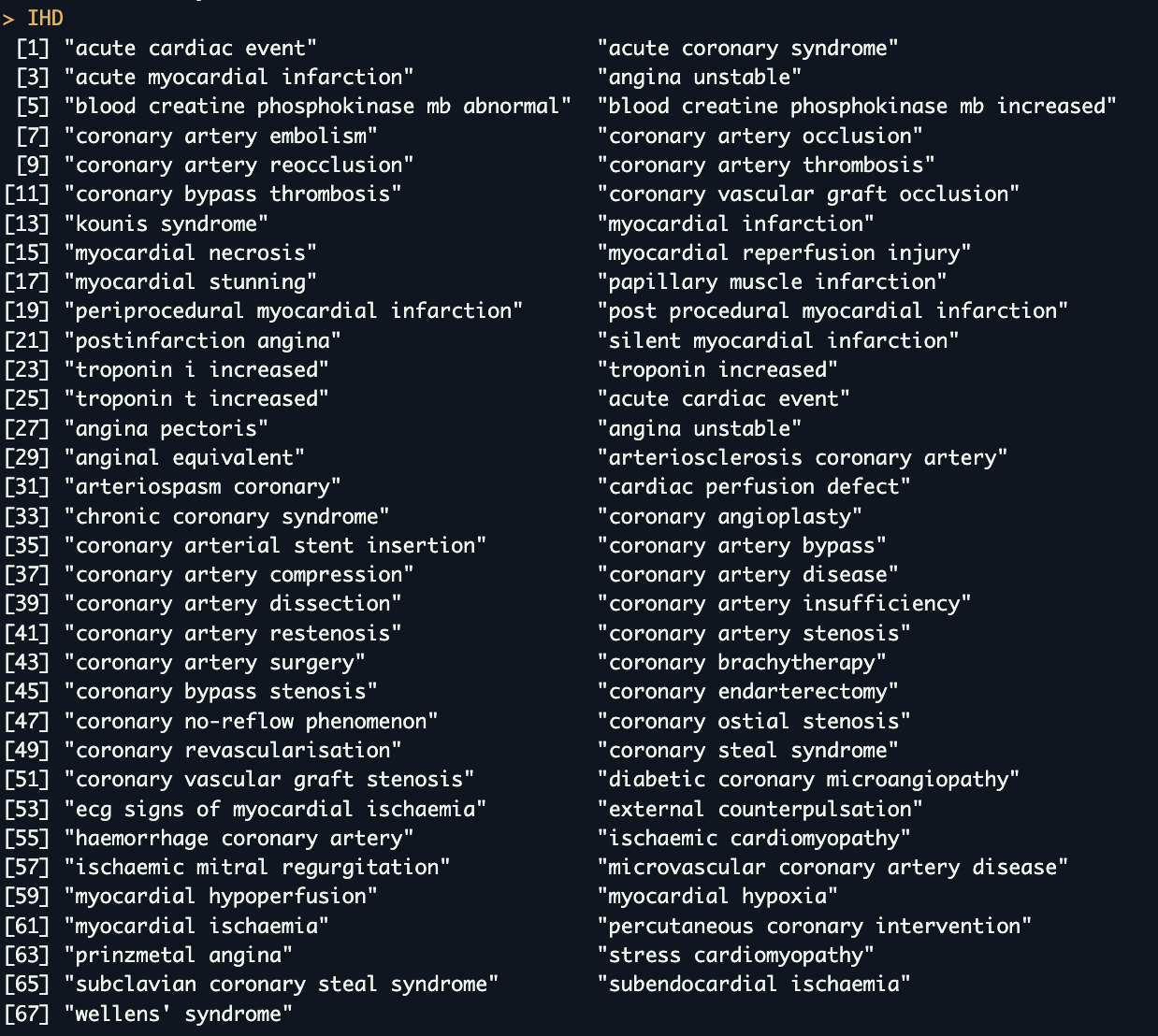


1. Cardiac failure (SMQ)


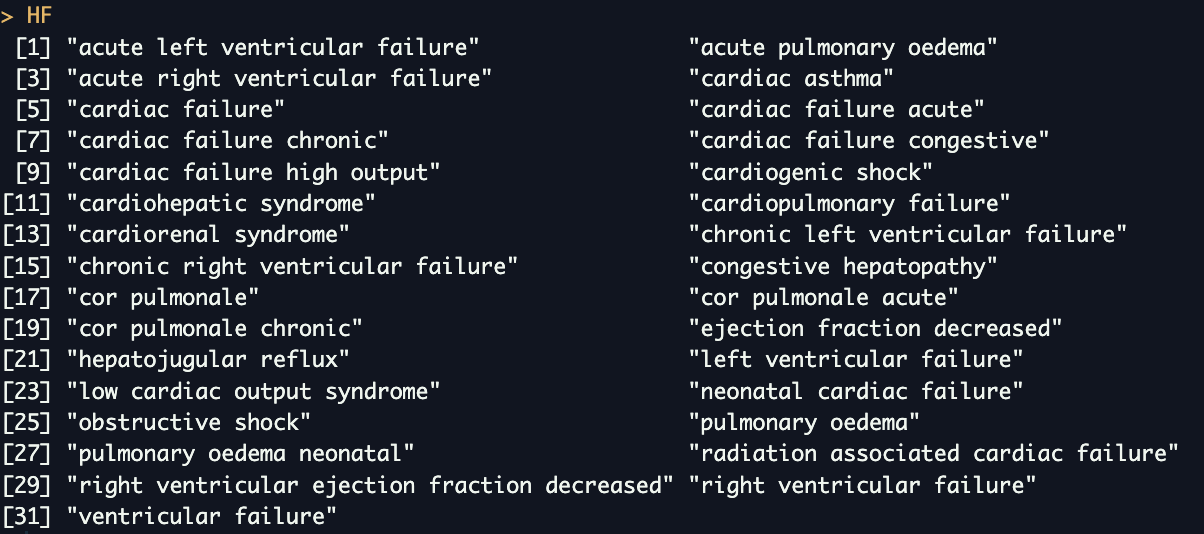


**Table S3**

Baseline characteristics of the patients enrolled in the studies included in the meta-analysis.

| **Study** | **Design** | **Indication for treatment** | **Intervention** | **Number of patients** | **Age (years)** | **Sex (male)** | **HTN** | **Diabetes** | **Previuous MI** | **PAD** | **LVEF** | **Betablockers** |
| --- | --- | --- | --- | --- | --- | --- | --- | --- | --- | --- | --- | --- |
|  |  |  |  |  |  |  |  |  |  |  |  |  |
| **BEAUTIFUL**  **(Fox et al., 2008)** | RCT | IHD and LVEF<40% | Iva vs. Pla | 5477 vs. 5430 | 65,2 (8,5) | 9047 (83%) | 7720 (71%) | 4036 (37%) | 9645 (88%) | 1440 (13%) | 32,4% (5,5%) | 9487 (87%) |
| **SHIFT**  **(Swedberg et al., 2010)** | RCT | Symptomatic HF and LVEF ≤35% | Iva vs. Pla | 3241 vs. 3264 | 60,4 (11,4) | 4970 (76%) | 4314 (66%) | 1979 (30%) | 3666 (56%) | NA | 29,0% (5%) | 5820 (89%) |
| **SIGNIFY**  **(Fox et al., 2014)** | RCT | IHD | Iva vs. Pla | 9539 vs. 9544 | 65,0 (7,3) | 13839 (72%) | 16466 (86%) | 8230 (43%) | 14002 (73%) | 4016 (21%) | 56,4 (8,5%) | 15878 (83%) |

HF, heart failure; HTN, hypertension; IHD, ischemic heart disease; LVEF, left ventricular ejection fraction; MI, myocardial infarction; PAD, peripheral artery disease

**Table S4**

Studies included in the meta-analysis and reported events retrieved from ClinicalTrials.gov.

| Study | Design | Year | Intervention | Number of patients | Ivabradine | | Placebo | | Median follow-up |
| --- | --- | --- | --- | --- | --- | --- | --- | --- | --- |
|  |  |  |  |  | **AF** | **stroke** | **AF** | **stroke** |  |
| BEAUTIFUL^7^ | RCT | 2008 | Iva vs. Pla | 5477 vs. 5438 | 334 | 107 | 310 | 119 | 19 months |
| SHIFT^9^ | RCT | 2010 | Iva vs. Pla | 3241 vs. 3264 | 309 | 68 | 238 | 94 | 22.9 months |
| SIGNIFY^10^ | RCT | 2014 | Iva vs. Pla | 9539 vs. 9544 | 520 | 213 | 374 | 204 | 27.8 months |

**Table S5**

| AE | Population | Drug | N. reports | IC (95% CI) |
| --- | --- | --- | --- | --- |
| Atrial Fibrillation_PT | HF | Beta_blockers   - Atenolol - Bisoprolol - Carvedilol - Metoprolol - Nadolol | 839  51  302  246  290  1 | 0.64 (0.53 – 0.72)  1.32 (0.85 – 1.65)  0.75 (0.56 – 0.88)  0.19 (-0.02 – 0.34)  1.05 (0.86 – 1.19)  -0.26 (-4.04 – 1.43) |
|  |  | Ivabradine | 56 | 0.76 (0.31 – 1.08) |
|  | IHD | Beta_blockers   - Atenolol - Bisoprolol - Carvedilol - Metoprolol - Nadolol | 596  69  179  96  286  1 | 0.42 (0.29 – 0.52)  0.49 (0.04 – 0.72)  0.51 (0.26 – 0.68)  0.13 (-0.21 – 0.37)  0.57 (0.38 – 0.71)  -0.43 (-4.22 – 1.26) |
|  |  | Ivabradine | 15 | 0.40 (-0.47 – 1.00) |
|  | IHD_HF | Beta_blockers   - Atenolol - Bisoprolol - Carvedilol - Metoprolol - Nadolol | 1344  114  453  314  540  2 | 0.53 (0.44 – 0.60)  0.55 (0.24 – 0.77)  0.69 (0.53 – 0.80)  0.29 (0.10 – 0.42)  0.68 (0.54 – 0.78)  -0.45 (-3.04 – 0.94) |
|  |  | Ivabradine | 66 | 0.84 (0.43 – 1.14) |
|  | IHD_HF post-approval | Beta_blockers   - Atenolol - Bisoprolol - Carvedilol - Metoprolol - Nadolol | 824  52  340  164  307  2 | 0.57 (0.46 – 0.65)  0.94 (0.48 – 1.27)  0.76 (0.58 – 0.89)  0.07 (-0.19 – 0.25)  0.72 (0.53 – 0.86)  0.35 (-2.24 – 1.74) |
|  |  | Ivabradine | 61 | 0.92 (0.49 – 1.22) |
|  |  |  |  |  |
| Stroke | HF | Beta_blockers   - Atenolol - Bisoprolol - Carvedilol - Metoprolol - Nadolol | 740  28  303  274  176  0 | 0.45 (0.33 – 0.54)  0.46 (-0.17 – 0.9)  0.74 (0.55 – 0.88)  0.34 (0.14 – 0.48)  0.32 (0.07 – 0.5)  -1.85 (-12.17 – 0.13) |
|  |  | Ivabradine | 34 | 0.04 (-0.54 – 0.44) |
|  | IHD | Beta_blockers   - Atenolol - Bisoprolol - Carvedilol - Metoprolol - Nadolol | 1346  155  361  287  619  2 | 0.12 (0.03 – 0.19)  0.13 (-0.13 – 0.32)  0.05 (-0.13 – 0.17)  0.23 (0.04 – 0.38)  0.21 (0.08 – 0.31)  -0.92 (-3.51 – 0.47) |
|  |  | Ivabradine | 20 | -0.63 (-1.38 - -0.10) |
|  | IHD_HF | Beta_blockers   - Atenolol - Bisoprolol - Carvedilol - Metoprolol - Nadolol | 1954  178  603  512  759  2 | 0.25 (0.18 – 0.31)  0.37 (0.12 – 0.55)  0.28 (0.15 – 0.38)  0.17 (0.03 – 0.28)  0.35 (0.23 – 0.44)  -1.17 (-3.77 – 0.22) |
|  |  | Ivabradine | 52 | -0.31 (-0.77 – 0.02) |
|  | IHD_HF post-approval | Beta_blockers   - Atenolol - Bisoprolol - Carvedilol - Metoprolol - Nadolol | 1087  44  456  303  330  0 | 0.26 (0.16 – 0.33)  -0.01 (-0.51 – 0.35)  0.47 (0.32 – 0.58)  0.24 (0.05 – 0.38)  0.11 (-0.07 – 0.25)  -2.53 (-12.86 – -0.55) |
|  |  | Ivabradine | 40 | -0.39 (-0.92 – -0.01) |

**Table S6**

| AE | Population | Drug | N. reports | IC (95% CI) |
| --- | --- | --- | --- | --- |
| Atrial Fibrillation_SMQ | HF | Beta_blockers   - Atenolol - Bisoprolol - Carvedilol - Metoprolol - Nadolol | 1016  58  365  332  336  2 | 0.65 (0.55 – 0.72)  1.24 (0.81 – 1.56)  0.75 (0.58 – 0.88)  0.36 (0.17 – 0.49)  1.00 (0.82 – 1.13)  0.28 (-2.31 – 1.67) |
|  |  | Ivabradine | 81 | 1.02 (0.65 – 1.29) |
|  | IHD | Beta_blockers   - Atenolol - Bisoprolol - Carvedilol - Metoprolol - Nadolol | 773  88  225  141  369  3 | 0.45 (0.33 – 0.54)  0.44 (0.09 – 0.70)  0.49 (0.27 – 0.65)  0.34 (0.06 – 0.54)  0.60 (0.42 – 0.72)  0.53 (-1.54 – 1.73) |
|  |  | Ivabradine | 18 | 0.33 (-0.46 – 0.88) |
|  | IHD_HF | Beta_blockers   - Atenolol - Bisoprolol - Carvedilol - Metoprolol - Nadolol | 1666  139  544  426  664  4 | 0.55 (0.47 – 0.61)  0.54 (0.26 – 0.74)  0.66 (0.51-0.76)  0.43 (0.27 – 0.55)  0.68 (0.55 – 0.77)  0.14 (-1.62 – 1.22) |
|  |  | Ivabradine | 93 | 1.04 (0.70 – 1.29) |
|  | IHD_HF post-approval | Beta_blockers   - Atenolol - Bisoprolol - Carvedilol - Metoprolol - Nadolol | 1003  63  412  218  371  2 | 0.60 (0.50 – 0.68)  0.96 (0.55 – 1.26)  0.79 (0.62 – 0.90)  0.23 (0 – 0.39)  0.74 (0.57 – 0.87)  0.16 (-2.43 – 1.55) |
|  |  | Ivabradine | 88 | 1.20 (0.84 – 1.45) |

**Figure S1**

These conceptual DAGs are proposed as a framework to interpret the observed association between ivabradine exposure and AF reporting. Both diagrams indicate a positive association between ivabradine and AF due to confounding by indication, as the diseases for which ivabradine is prescribed carry a higher risk of AF.

Under H₀ (biological causation), ivabradine exerts a direct pro-arrhythmic effect (D → E_1_), leading to a genuine increase in AF incidence and occurrence of ischemic cerebrovascular events.

Under H₁ (enhanced detection), the apparent rise in AF reports may stem from a higher likelihood of diagnosis (D → M_1_): by lowering heart rate during sinus rhythm, ivabradine may amplify symptom perception when AF occurs, thereby facilitating arrhythmia recognition and prompting earlier initiation of anticoagulation, with the potential to reduce ischemic cerebrovascular events.


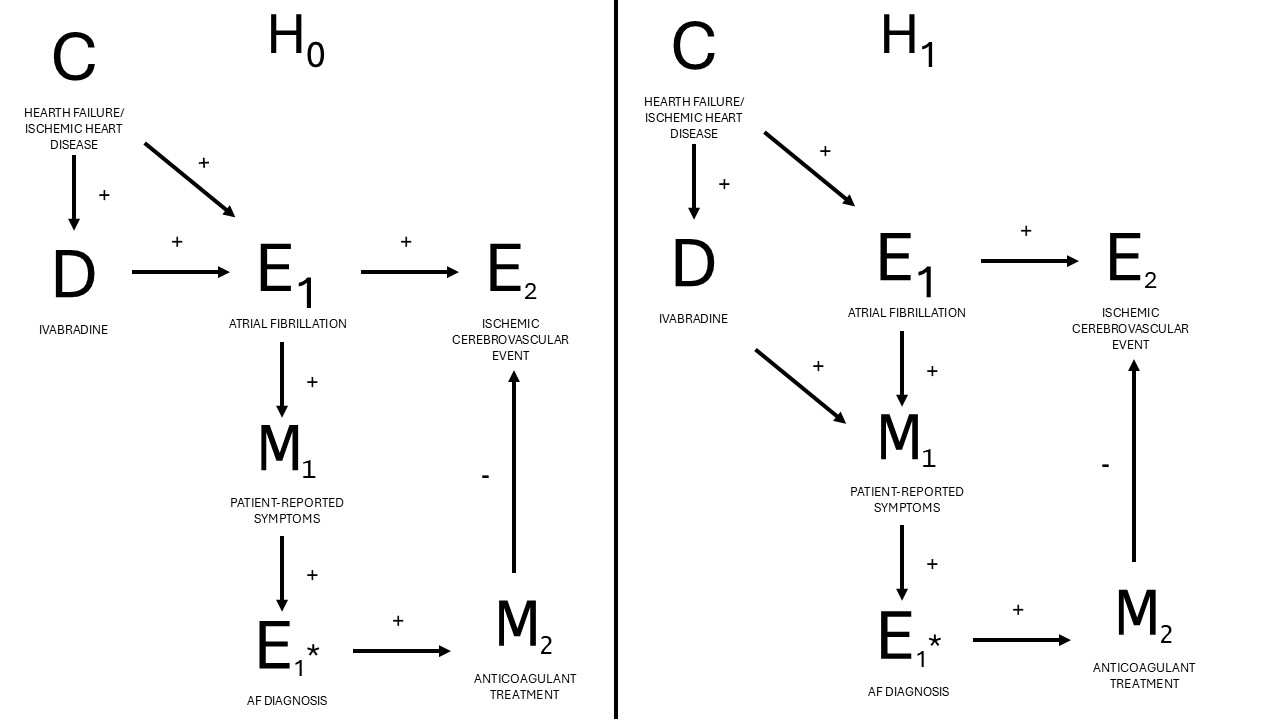


**Figure S2**

Egger’s plot assessing publication bias among included studies


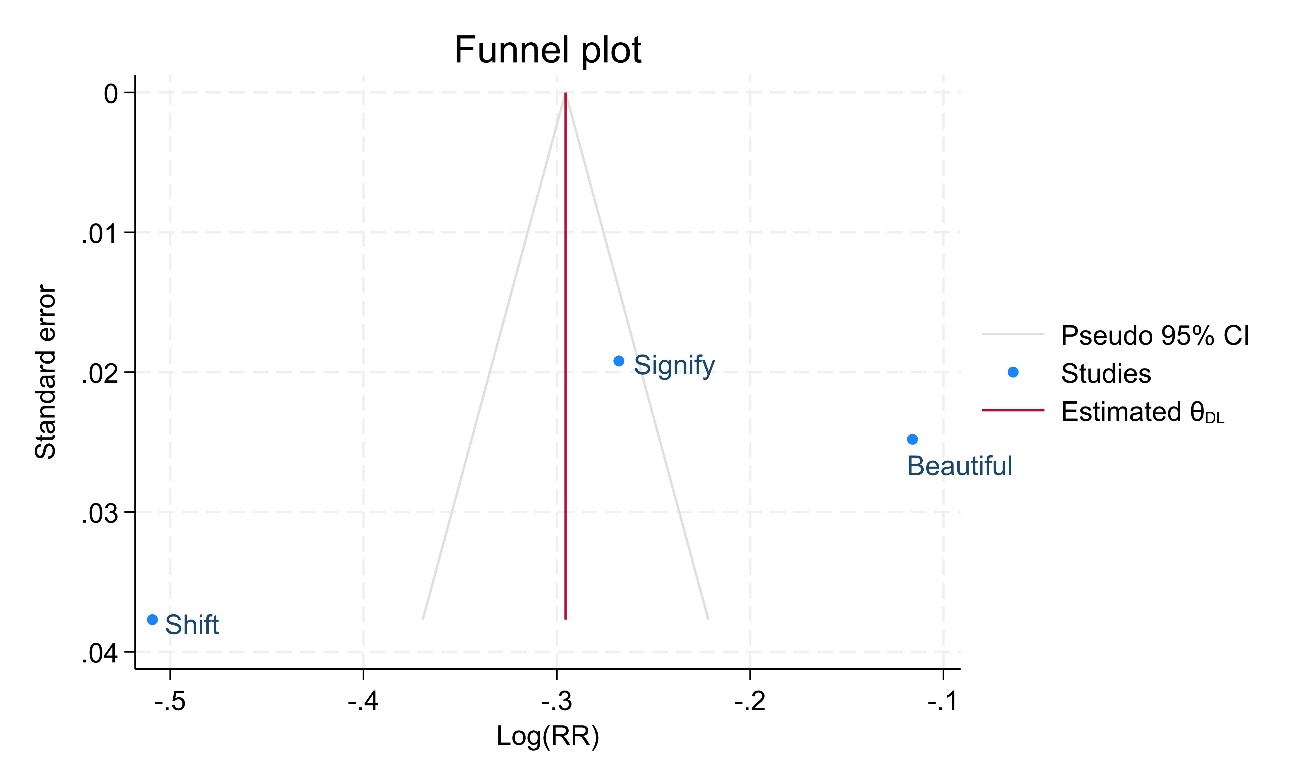


**Figure S3**

Forest plot showing the ratio of ischemic cerebrovascular events to atrial fibrillation with data retrieved from ClinicalTrials.gov.


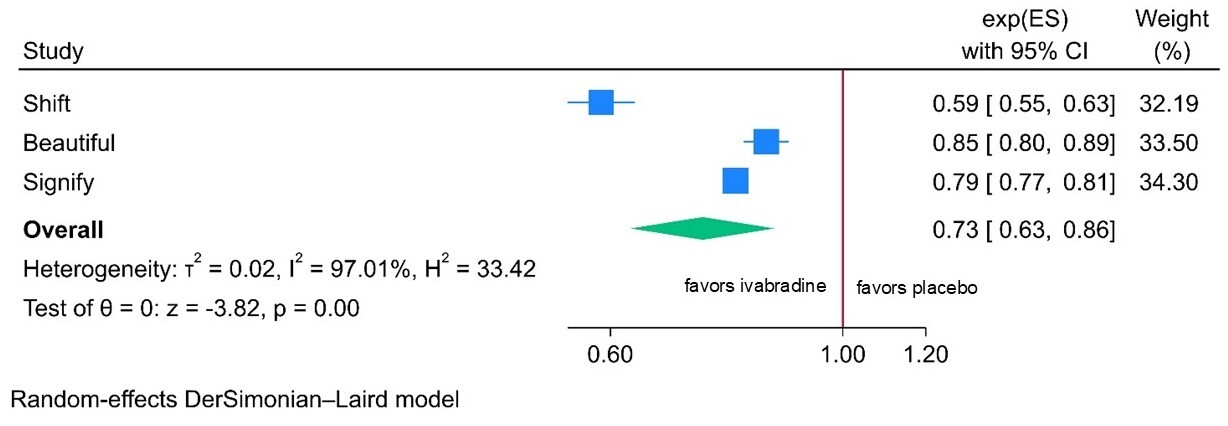

Supplement: Supplementary file 1 [file Supplementaryfile1.docx]
